# Alcohol consumption and mortality among stroke survivors: A NHANES observational cohort study with mediation analysis

**DOI:** 10.1097/MD.0000000000047514

**Published:** 2026-02-06

**Authors:** Xiaoxiu Zhu, Qianqian Zhang

**Affiliations:** a Emergency Department, Suzhou Benq Hospital, Suzhou, Jiangsu, China.

**Keywords:** alcohol consumption, all-cause mortality, cardiovascular mortality, inflammatory biomarkers, stroke survivor

## Abstract

The long-term effects of alcohol consumption among stroke survivors are unclear. This study investigated the association between alcohol intake and all-cause and cardiovascular mortality, and whether inflammatory markers mediate this relationship. A total of 793 stroke survivors from the National Health and Nutrition Examination Survey 2005 to 2016 were classified by alcohol intake frequency. Mortality data were obtained from the National Death Index. Cox models estimated hazard ratios for mortality. Mediation analysis examined inflammatory biomarkers (neutrophil-to-lymphocyte ratio, platelet-to-lymphocyte ratio, neutrophil-albumin ratio, white blood cell count). During follow-up, 313 participants died (86 cardiovascular deaths). Mild and moderate drinking were associated with reduced all-cause mortality compared to former drinkers (hazard ratio = 0.711 and 0.657, respectively; *P* < .05). No significant association was found with cardiovascular mortality. A J-shaped association was observed between alcohol use and all-cause death. Inflammatory markers showed minimal, nonsignificant mediation (≤8.2%). Light-to-moderate alcohol consumption was statistically associated with lower all-cause mortality among stroke survivors, while no significant association was observed for cardiovascular mortality. These findings represent observational associations based on the available data.

## 1. Introduction

Stroke constitutes 1 of the foremost causes of death and disability across the globe. According to the Global Burden of Disease Study, over 12 million new stroke cases occurred globally in 2019, resulting in more than 6.5 million deaths and 143 million disability-adjusted life years lost.^[1,2]^ Roughly 795,000 individuals in the U.S. are affected by stroke annually, with nearly 25% being recurrent events.^[3,4]^ As survival rates improve due to advances in acute stroke management, stroke survivors now represent a growing high-risk population requiring long-term care and secondary prevention strategies.^[5]^ Identifying modifiable behavioral factors that impact long-term mortality, particularly all-cause and cardiovascular-specific death, is a key priority in poststroke population health.^[6]^

Alcohol consumption is 1 such behavioral factor with complex and often debated associations with health outcomes. A growing body of evidence suggests a J-shaped relationship between alcohol intake and all-cause mortality, in which light-to-moderate drinking appears protective compared to abstinence or heavy consumption.^[7]^ However, these findings are subject to methodological challenges, including reverse causation and misclassification bias. For instance, the “sick quitter” effect, where individuals abstain due to preexisting health conditions, may artificially inflate mortality risks among nondrinkers.^[8,9]^ Moreover, evidence in stroke survivors remains scarce, despite their distinct baseline risk profiles, medication regimens, and vascular vulnerability compared to the general population.

Emerging mechanistic insights suggest that systemic inflammation may play a pivotal role in mediating the health effects of alcohol. Chronic alcohol use is known to influence immune responses through oxidative stress, cytokine activation, and alterations in leukocyte profiles.^[10,11]^ Inflammatory biomarkers such as the neutrophil-to-lymphocyte ratio (NLR), platelet-to-lymphocyte ratio (PLR), neutrophil-albumin ratio (NPAR), and white blood cell (WBC) count have been linked to increased cardiovascular risk and mortality in both general and stroke-specific populations.^[12,13]^ However, it remains unclear whether alcohol consumption affects mortality among stroke survivors via modulation of these inflammatory mediators.

To fill these gaps in knowledge, we performed a population-based analysis based on data from the National Health and Nutrition Examination Survey (NHANES) spanning 2005 to 2016. Our objective was to explore the relationship between alcohol consumption frequency and all-cause and cardiovascular mortality among adults with a history of stroke. We also evaluated whether selected inflammatory biomarkers mediate these associations. By leveraging a large, nationally representative real-world dataset and applying robust statistical approaches, the present study seeks to define dose-response relationships and uncover biologically plausible pathways relevant to stroke recovery and long-term prognosis.

## 2. Methods

### 2.1. Data source and study population

**Figure 1.**
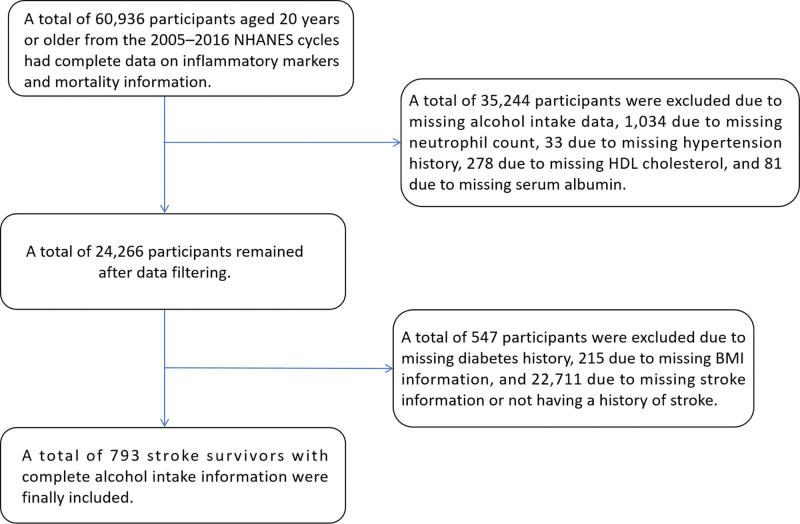
Flowchart illustrating the selection of stroke survivors from the NHANES 2005 to 2016 database. NHANES: National Health and Nutrition Examination Survey, BMI: body mass index, HDL = high-density lipoprotein.

We analyzed NHANES, a crosssectional, CDC-conducted program representative of the national population. 6 consecutive survey cycles from 2005 to 2016 were combined to construct the analytic dataset. Eligibility criteria included age ≥20 years ≥20 years, self-reported a history of stroke, and having complete data on alcohol consumption, inflammatory markers, and mortality outcomes. Exclusion criteria included pregnancy, age <20 years, or missing information on key variables, including alcohol intake, inflammatory markers, covariates, or survival status. After applying these criteria, 793 stroke survivors were retained for the final analysis, as illustrated in the participant flowchart (Fig. 1).

## 3. Ethical approval

NHANES data are publicly available and fully de-identified. The NHANES protocol was approved by the National Center for Health Statistics (NCHS) Research Ethics Review Board, and all participants provided written informed consent. Because this study involved secondary analysis of anonymized public-use data, additional institutional review board approval and informed consent were not required.

### 3.1. Classification of alcohol consumption

Alcohol intake was assessed through structured questionnaires administered during household interviews. Based on alcohol use frequency over the past year, participants were classified into 4 mutually exclusive groups: “Former” drinkers (previous alcohol use but no consumption in the past 12 months), “Light” drinkers (no more than 1 drink per day for women or 2 drinks per day for men), “Moderate” drinkers (no more than 3 drinks per day for women or 4 drinks per day for men), and “Heavy” drinkers (4 or more drinks per day for women or 5 or more drinks per day for men).^[14]^

### 3.2. Mortality outcomes

Mortality status was determined by linkage to the National Death Index, with follow-up through December 31, 2019. The primary outcomes were all-cause mortality (death from any cause) and cardiovascular mortality, defined by ICD-10 codes I00–I09, I11, I13, I20–I51, and I60–I69, covering both heart disease and cerebrovascular events. These variables were provided and validated by the National Center for Health Statistics for use in longitudinal NHANES-based mortality studies.

### 3.3. Inflammatory markers

Four systemic inflammatory markers were selected based on their established relevance in vascular disease outcomes: the NLR, PLR, NPAR, and WBC count. NLR, PLR, and NPAR were calculated as follows: NLR = neutrophil count/lymphocyte count; PLR = platelet count/lymphocyte count; NPAR = neutrophil count/serum albumin. WBC count was measured directly in units of 10^9^/L. All biomarkers were obtained from fasting blood samples collected at NHANES mobile examination centers using standardized laboratory procedures.

### 3.4. Covariates

Potential confounders were selected a priori based on existing literature and included demographic, behavioral, and clinical characteristics: age, sex, race/ethnicity, educational attainment, smoking status (never, former, current), body mass index (BMI), history of diabetes or hypertension, total cholesterol, and high-density lipoprotein cholesterol. All variables were obtained via structured interviews, physical examinations, or laboratory assessments following standardized NHANES protocols.

### 3.5. Statistical analysis

Baseline characteristics were summarized across alcohol consumption groups using means with standard deviations or medians with interquartile ranges for continuous variables, and frequencies with percentages for categorical variables. Group differences were assessed via 1-way ANOVA or Kruskal-Wallis tests for continuous data, and chi-square tests for categorical variables. Survival probabilities were estimated using Kaplan-Meier curves, and differences were evaluated using the log-rank test. To evaluate the association between alcohol consumption and mortality risk, Cox proportional hazards models were employed. Three models were fitted: Model 1 was unadjusted; Model 2 adjusted for age, sex, race/ethnicity, BMI, smoking status, and education level; and Model 3 further adjusted for diabetes, hypertension, TC, and high-density lipoprotein cholesterol. The proportional hazards assumption was tested using Schoenfeld residuals, and no significant violations were observed. Restricted cubic spline (RCS) models were used to assess potential nonlinear dose-response patterns, with alcohol frequency treated as a continuous predictor.

To examine indirect pathways, mediation analyses estimated the effects of inflammatory biomarkers (NLR, PLR, NPAR, and WBC) on the alcohol-mortality relationship. Both single and multiple mediator models were applied, and the proportion mediated was calculated. All analyses were conducted using R software (version 4.3.3; Vienna, Austria), and statistical significance was defined as a 2-sided *P*-value < 0.05.

## 4. Results

### 4.1. Baseline characteristics of the study population

Baseline characteristics of the 793 stroke survivors are summarized in Table 1. The median follow-up time of the cohort was 6.67 years (interquartile range 4.25–9.83), contributing a total of 5578 person-years of observation. A total of 313 participants experienced all-cause mortality during follow-up. Compared with survivors, decedents were significantly older (72.33 ± 9.55 vs 60.44 ± 13.42, *P* < .001), more likely to be male (63.6% vs 50.0%, *P* = .0002), and predominantly non-Hispanic (86.3% vs 76.7%, *P* = .0035). They also had a lower mean BMI (29.03 ± 6.76 vs 30.62 ± 6.99, *P* = .0015) and were less likely to have received higher education (*P* = .0486). The prevalence of hypertension was significantly higher in the mortality group (80.2% vs 72.7%, *P* = .0206). In terms of inflammatory biomarkers, decedents exhibited significantly elevated levels of NLR (*P* < .001), PLR (*P* = .0245), and NPAR (*P* = .0016). Alcohol consumption patterns also differed significantly across survival status (*P* < .001), with former drinkers overrepresented among deaths (57.5%), and moderate drinkers accounting for a smaller proportion (10.5%).

**Table 1:** Baseline characteristics of stroke survivors according to all-cause mortality status.

Variable	Dead (n = 313)	Alive (n = 480)	*t*/*χ*^2^	*P* value
Age, mean ± SD (yr)	72.33 ± 9.55	60.44 ± 13.42	−14.56	<.001
Gender, n (%)			13.59	.0002
Male	199 (63.6%)	240 (50.0%)		
Female	114 (36.4%)	240 (50.0%)		
Race/ethnicity, n (%)			11.33	.0035
Hispanic	34 (10.9%)	84 (17.5%)		
Non-Hispanic	270 (86.3%)	368 (76.7%)		
Other	9 (2.9%)	28 (5.8%)		
BMI, mean ± SD (kg/m^2^)	29.03 ± 6.76	30.62 ± 6.99	3.19	.0015
Smoking status, n (%)			3.74	.0531
Yes	231 (73.8%)	322 (67.1%)		
No	82 (26.2%)	158 (32.9%)		
Education level, n (%)			3.89	.0486
High school and below	204 (65.2%)	278 (57.9%)		
University and above	109 (34.8%)	202 (42.1%)		
Diabetes, n (%)			0.32	.5697
Yes	106 (33.9%)	152 (31.7%)		
No	207 (66.1%)	328 (68.3%)		
Hypertension, n (%)			5.36	.0206
Yes	251 (80.2%)	349 (72.7%)		
No	62 (19.8%)	131 (27.3%)		
Total cholesterol (mmol/L)	4.70 ± 1.37	4.82 ± 1.11	1.34	.18
HDL-C (mmol/L)	1.33 ± 0.47	1.32 ± 0.41	−0.35	.7254
Alcohol intake, n (%)			28.63	<.001
Former	180 (57.5%)	195 (40.6%)		
Mild	58 (18.5%)	146 (30.4%)		
Moderate	33 (10.5%)	84 (17.5%)		
Heavy	42 (13.4%)	55 (11.5%)		
NPAR, mean ± SD	0.12 ± 0.05	0.11 ± 0.04	−3.17	.0016
PLR, mean ± SD	140.6 ± 77.09	129.03 ± 59.46	−2.26	.0245
NLR, mean ± SD	2.99 ± 2.34	2.31 ± 1.14	−4.8	<.001
WBC count (×10^9^/L)	7.74 ± 3.57	7.40 ± 2.11	−1.49	.1359

BMI = body mass index, HDL-C = high-density lipoprotein cholesterol, NLR = neutrophil-to-lymphocyte ratio, NPAR = neutrophil-albumin ratio, PLR = platelet-to-lymphocyte ratio, SD = standard deviations, WBC = white blood cell count.

Among the 86 participants who died from cardiovascular causes, significant differences were observed in fewer variables (Table 2). These individuals were older (72.38 ± 10.20 vs 64.25 ± 13.44, *P* < .001), more likely to be male (68.6% vs 53.7%, *P* = .0124), and more frequently non-Hispanic (90.7% vs 79.2%, *P* = .0344) compared to survivors. A higher prevalence of hypertension was additionally noted (84.9% vs 74.5%, *P* = .048). Among inflammatory indices, only NLR remained significantly elevated in the cardiovascular mortality group (*P* = .0207), while PLR, NPAR, and WBC count showed no significant differences.

**Table 2:** Baseline characteristics of stroke survivors according to cardiovascular mortality status.

Variable	CVD death (n = 86)	Survivors (n = 707)	*t*/*χ*^2^	*P* value
Age, mean ± SD (yr)	72.38 ± 10.20	64.25 ± 13.44	6.72	<.001
Gender, n (%)			6.26	.0124
Male	59 (68.6%)	380 (53.7%)		
Female	27 (31.4%)	327 (46.3%)		
Race/ethnicity, n (%)			6.74	.0344
Hispanic	7 (8.1%)	111 (15.7%)		
Non-Hispanic	78 (90.7%)	560 (79.2%)		
Other	1 (1.2%)	36 (5.1%)		
BMI, mean ± SD (kg/m^2^)	30.51 ± 7.88	29.93 ± 6.82	−0.66	.5116
Smoking status, n (%)			0.77	.3805
Yes	64 (74.4%)	489 (69.2%)		
No	22 (25.6%)	218 (30.8%)		
Education level, n (%)			2.51	.1132
High school and below	45 (52.3%)	437 (61.8%)		
University and above	41 (47.7%)	270 (38.2%)		
Diabetes, n (%)			0.38	.539
Yes	31 (36.0%)	227 (32.1%)		
No	55 (64.0%)	480 (67.9%)		
Hypertension, n (%)			3.91	.048
Yes	73 (84.9%)	527 (74.5%)		
No	13 (15.1%)	180 (25.5%)		
Total cholesterol (mmol/L)	4.64 ± 1.39	4.79 ± 1.20	0.95	.3437
HDL-C (mmol/L)	1.26 ± 0.42	1.33 ± 0.44	1.4	.1642
Alcohol intake, n (%)			3.86	.2772
Former	49 (57.0%)	326 (46.1%)		
Mild	19 (22.1%)	185 (26.2%)		
Moderate	9 (10.5%)	108 (15.3%)		
Heavy	9 (10.5%)	88 (12.4%)		
NPAR, mean ± SD	0.11 ± 0.04	0.11 ± 0.04	−0.7	.4876
PLR, mean ± SD	142.64 ± 59.57	132.5 ± 67.99	−1.47	.1452
NLR, mean ± SD	2.93 ± 1.41	2.54 ± 1.78	−2.34	.0207
WBC count (×10^9^/L)	7.37 ± 2.06	7.55 ± 2.86	0.74	.4605

BMI = body mass index, CVD = cardiovascular diseases, HDL-C = high-density lipoprotein cholesterol, NLR = neutrophil-to-lymphocyte ratio, NPAR = neutrophil-albumin ratio, PLR = platelet-to-lymphocyte ratio, SD = standard deviations, WBC = white blood cell count.

### 4.2. Survival outcomes by alcohol consumption frequency

**Figure 2.**
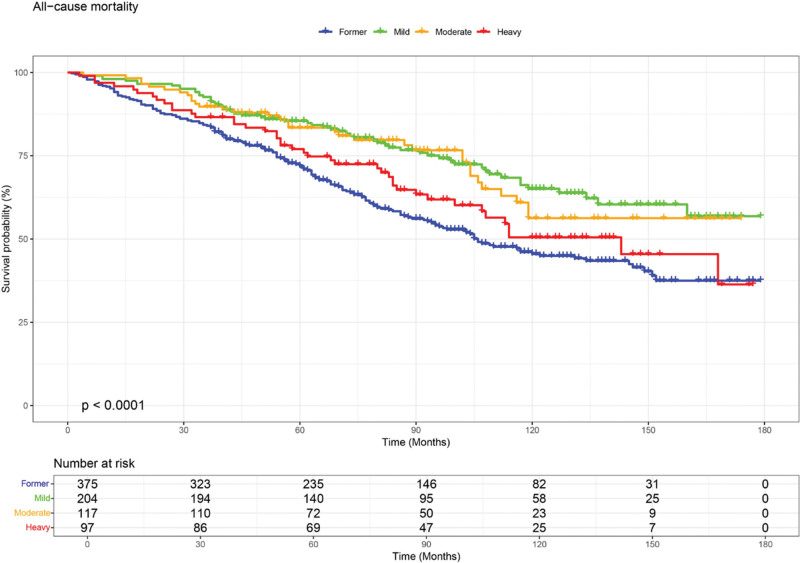
Kaplan-Meier survival curves showing all-cause mortality among stroke survivors by alcohol consumption category. Groups included former (blue), mild (green), moderate (orange), and heavy (red) drinkers. Survival differed significantly across categories (log-rank *P* < .0001), with mild drinkers demonstrating the highest survival and former drinkers the lowest. The number of participants at risk is indicated below the x-axis.

**Figure 3.**
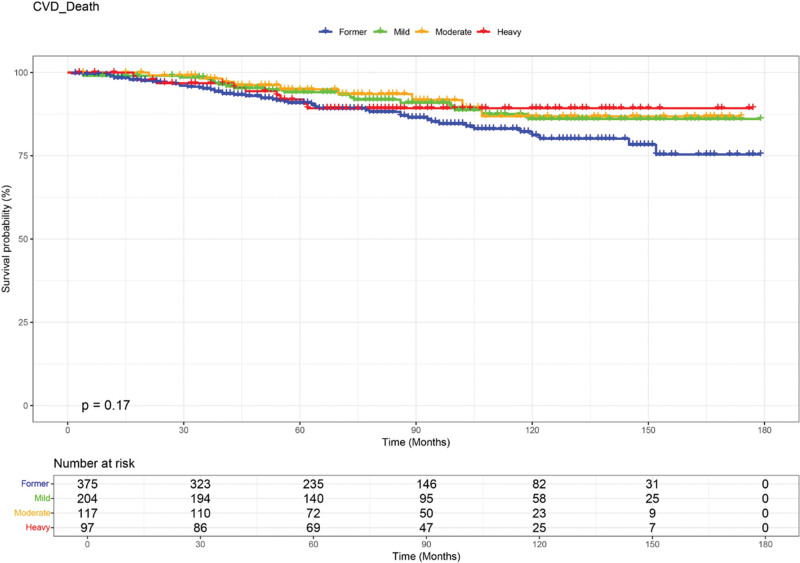
Kaplan-Meier survival curves for cardiovascular mortality among stroke survivors stratified by alcohol consumption. No statistically significant differences in cardiovascular survival were detected among groups (log-rank *P* = .17). Risk counts by group are presented below the x-axis. CVD = cardiovascular diseases.

Kaplan-Meier analysis revealed significant differences in all-cause mortality across alcohol consumption groups (log-rank *P* < .0001, Fig. 2). The mild drinking group demonstrated the highest cumulative survival probability, followed by the moderate and heavy groups, while former drinkers consistently showed the lowest survival rate. Group separation emerged early during follow-up and remained stable throughout the observation period, indicating sustained differences in survival patterns by drinking frequency. In contrast, cardiovascular mortality did not differ significantly across groups (log-rank *P* = .17, Fig. 3). Survival curves were closely aligned over time, and although former drinkers exhibited a trend toward lower survival, these differences did not reach statistical significance.

### 4.3. Association between alcohol consumption and mortality risk

For all-cause mortality (Table 3), both mild and moderate alcohol consumption were consistently associated with a significantly reduced risk across all models. In the unadjusted model (Model 1), the hazard ratio (HR) was 0.540 (95% confidence interval (CI): 0.40–0.74, *P* = .0002) for mild drinking and 0.470 (95% CI: 0.32–0.69, *P* = .0004) for moderate drinking. After adjustment for demographic and lifestyle covariates (Model 2), these associations remained statistically significant, with HRs of 0.722 (95% CI: 0.53–0.99, *P* = .0425) and 0.669 (95% CI: 0.45–0.99, *P* = .0368), respectively. In the fully adjusted model (Model 3), which included clinical comorbidities and lipid profiles, mild and moderate alcohol use remained protective, with HRs of 0.711 (95% CI: 0.52–0.98; *P* = .0388) and 0.657 (95% CI: 0.44–0.99; *P* = .0351), respectively. For heavy drinking, the HRs across all models were not statistically significant, although Model 3 suggested a possible trend toward lower risk (HR = 0.707; 95% CI: 0.48–1.05; *P* = .0833).

**Table 3:** Multivariable Cox regression analysis of the association between alcohol consumption and all-cause mortality.

Alcohol group	Model 1		Model 2		Model 3	
	HR (95% CI)	*P*	HR (95% CI)	*P*	HR (95% CI)	*P*
Former	1		1		1	
Mild	0.540 (0.40–0.74)	.0002	0.722 (0.53–0.99)	.0425	0.711 (0.52–0.98)	.0388
Moderate	0.470 (0.32–0.69)	.0004	0.669 (0.45–0.99)	.0368	0.657 (0.44–0.99)	.0351
Heavy	0.827 (0.57–1.19)	.400	0.729 (0.50–1.07)	.112	0.707 (0.48–1.05)	.0833

CI = confidence interval, HR = hazard ratio.

For cardiovascular mortality (Table 4), no statistically significant associations were observed in any model. In Model 1, the HRs were 0.510 (95% CI: 0.28–0.94, *P* = .0137) for mild, 0.426 (95% CI: 0.20–0.93, *P* = .104) for moderate, and 0.832 (95% CI: 0.41–1.69, *P* = .689) for heavy drinking. These associations lost statistical significance after adjustment. In Model 3, the HRs were 0.696 (95% CI: 0.37–1.30, *P* = .226), 0.611 (95% CI: 0.27–1.38, *P* = .306), and 0.699 (95% CI: 0.32–1.50, *P* = .376) for mild, moderate, and heavy drinking, respectively.

**Table 4:** Multivariable Cox regression analysis of the association between alcohol consumption and cardiovascular mortality.

Alcohol group	Model 1		Model 2		Model 3	
	HR (95% CI)	*P*	HR (95% CI)	*P*	HR (95% CI)	*P*
Former	1		1		1	
Mild	0.510 (0.28–0.94)	.0137	0.662 (0.36–1.23)	.149	0.696 (0.37–1.30)	.226
Moderate	0.426 (0.20–0.93)	.104	0.619 (0.28–1.37)	.32	0.611 (0.27–1.38)	.306
Heavy	0.832 (0.41–1.69)	.689	0.678 (0.32–1.42)	.299	0.699 (0.32–1.50)	.376

CI = confidence interval, HR = hazard ratio.

### 4.4. Dose-response relationship between alcohol consumption and mortality risk

**Figure 4.**
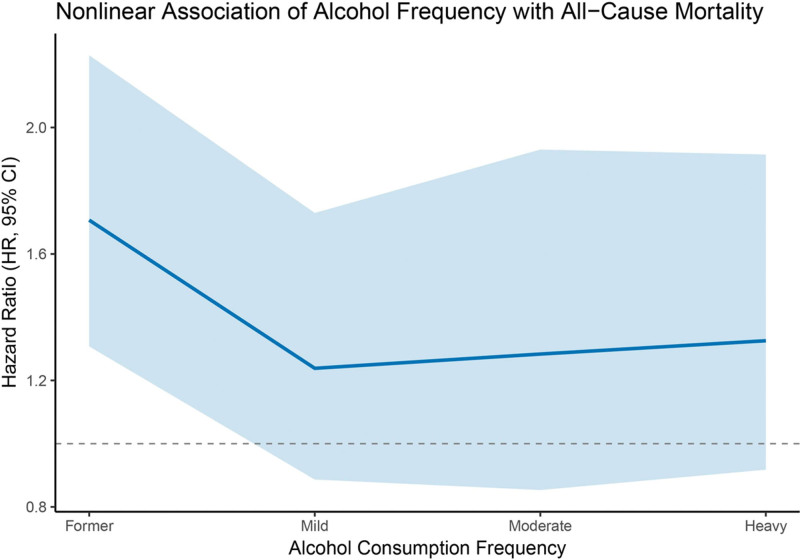
Restricted cubic spline (RCS) regression showing the nonlinear association between alcohol consumption frequency and all-cause mortality among stroke survivors. The former drinking group was used as the reference. The solid line represents the estimated HR, and the shaded area indicates the 95% CI. A J-shaped pattern was observed, with the lowest HR in mild drinkers and increased risk at both extremes. CI = confidence interval, HR = hazard ratio.

**Figure 5.**
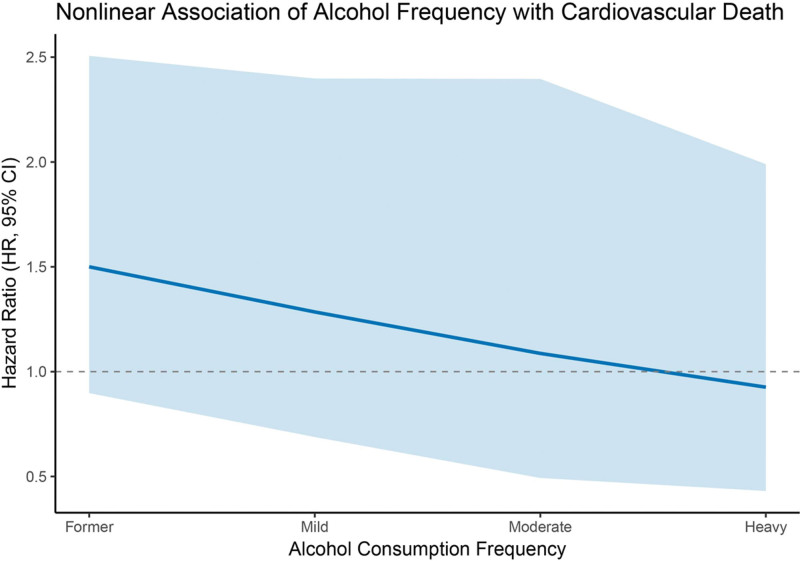
Restricted cubic spline (RCS) regression showing the nonlinear association between alcohol consumption frequency and cardiovascular mortality among stroke survivors. No statistically significant association was found. The solid line represents the estimated HR, and the shaded area indicates the 95% CI. CI = confidence interval, HR = hazard ratio.

The nonlinear associations between alcohol consumption frequency and mortality risk were assessed using RCS regression, with the former drinking group as the reference. As illustrated in Figure 4, a J-shaped relationship was observed for all-cause mortality, with the lowest HR occurring in the mild drinking group, followed by a gradual increase in risk with higher alcohol consumption. The 95% CI was narrowest for mild and moderate intake, indicating more stable estimates in these categories. In contrast, the CIs widened considerably for former and heavy drinkers, reflecting greater uncertainty at the distribution tails. In Figure 5, the spline analysis for cardiovascular mortality showed a generally decreasing trend in HR across increasing alcohol intake. However, the association was not statistically significant, and the 95% CIs remained wide throughout, suggesting limited precision in the estimates and no conclusive evidence of a dose-dependent effect.

### 4.5. Mediation effects of inflammatory biomarkers between alcohol consumption and mortality

Mediation analyses were performed to assess whether inflammatory markers mediated the association between alcohol consumption and all-cause or cardiovascular mortality. For all-cause mortality (Table 5), none of the indirect effects reached statistical significance across individual mediators. The HRs for the indirect paths were approximately null, with *P* values of .8933 (NLR), .7733 (PLR), .1600 (NPAR), and .0800 (WBC). The proportion of the total effect mediated ranged from 0.63% (PLR) to 7.67% (NPAR). In multiple mediator models, the indirect effects also remained nonsignificant (*P* = .3867 for NLR + PLR + NPAR; *P* = .2800 for NLR + PLR + NPAR + WBC), with corresponding mediated proportions of 7.59% and 8.20%, respectively. Notably, PLR showed a significant direct effect (HR = 1.0490, *P* = .0267), and the NLR + PLR + NPAR combination approached significance (*P* = .0533), suggesting partial but weak direct associations, not mediated through inflammatory pathways.

**Table 5:** Mediation analysis of inflammatory biomarkers in the association between alcohol consumption and all-cause mortality.

Mediator(s)	Indirect effect HR (95% CI)	*P*-value	Direct effect HR (95% CI)	*P*-value	Total effect HR (95% CI)	*P*-value	Proportion mediated (%)
NLR	1.0023 (0.91–1.10)	.8933	0.7113 (0.52–0.98)	.0388	0.7130 (0.52–0.98)	.0385	2.1
PLR	1.0044 (0.92–1.10)	.7733	0.7096 (0.52–0.98)	.039	0.7128 (0.52–0.98)	.0386	0.63
NPAR	1.0180 (0.98–1.06)	.16	0.7000 (0.51–0.97)	.0345	0.7128 (0.52–0.98)	.0386	7.67
WBC	1.0210 (0.99–1.06)	.08	0.6993 (0.51–0.97)	.0343	0.7135 (0.52–0.98)	.0389	6.58
NLR + PLR + NPAR	1.0176 (0.98–1.06)	.3867	0.7001 (0.51–0.97)	.0342	0.7131 (0.52–0.98)	.0386	7.59
NLR + PLR + NPAR + WBC	1.0203 (0.99–1.06)	.28	0.6989 (0.51–0.97)	.0341	0.7132 (0.52–0.98)	.0386	8.2

CI = confidence interval, HR = hazard ratio, NLR = neutrophil-to-lymphocyte ratio, NPAR = neutrophil-albumin ratio, PLR = platelet-to-lymphocyte ratio, WBC = white blood cell count.

For cardiovascular mortality (Table 6), neither individual nor combined inflammatory markers demonstrated significant indirect effects. *P* values for single mediators were all >.32, with proportion mediated values consistently <1%, and even negative for WBC (−3.44%). In multiple mediator models, the indirect path remained nonsignificant, with a mediated proportion of 1.14% (*P* = .933) without WBC, and –1.05% (*P* = .733) with WBC included. These results indicate negligible mediating roles of inflammation in the association between alcohol use and cardiovascular death.

**Table 6:** Mediation analysis of inflammatory biomarkers in the association between alcohol consumption and cardiovascular mortality.

Mediator(s)	Indirect effect HR (95% CI)	*P*-value	Direct effect HR (95% CI)	*P*-value	Total effect HR (95% CI)	*P*-value	Proportion mediated (%)
NLR	1.0002 (0.9963–1.0050)	.96	1.0336 (0.9795–1.0750)	.227	1.0339 (0.9795–1.0730)	.213	0.69
PLR	1.0003 (0.9975–1.0040)	.747	1.0326 (0.9847–1.0930)	.2	1.0330 (0.9871–1.0940)	.187	0.95
NPAR	1.0002 (0.9978–1.0030)	.947	1.0335 (0.9917–1.0840)	.16	1.0338 (0.9921–1.0840)	.16	0.71
WBC	0.9989 (0.9959–1.0010)	.32	1.0346 (0.9789–1.0890)	.28	1.0335 (0.9787–1.0890)	.28	–3.44
NLR + PLR + NPAR	1.0004 (0.9966–1.0070)	.933	1.0327 (0.9837–1.0820)	.227	1.0330 (0.9849–1.0840)	.173	1.14
NLR + PLR + NPAR + WBC	0.9997 (0.9928–1.0050)	.733	1.0334 (0.9873–1.0930)	.147	1.0330 (0.9870–1.0920)	.16	−1.

CI = confidence interval, HR = hazard ratio, NLR = neutrophil-to-lymphocyte ratio, NPAR = neutrophil–albumin ratio, PLR = platelet-to-lymphocyte ratio, WBC = white blood cell count.

## 5. Discussion

This nationally representative analysis investigated the association between alcohol consumption and mortality risk among stroke survivors using data from a nationally representative cohort. Several key findings emerged. First, both mild and moderate alcohol consumption were independently associated with a significantly lower risk of all-cause mortality compared to former drinkers, even after adjustment for demographic, clinical, and behavioral confounders. Second, no statistically significant associations were found between alcohol intake and cardiovascular mortality in any adjusted model. Third, restricted cubic spline analysis revealed a J-shaped dose-response relationship for all-cause mortality, while the relationship with cardiovascular mortality remained flat and nonsignificant. Lastly, mediation analyses showed that systemic inflammatory markers, including NLR, PLR, NPAR, and WBC, did not significantly mediate the observed associations.

These findings align with prior large-scale observational studies reporting a nonlinear, often J-shaped association between alcohol consumption and all-cause mortality. For example, Wood et al reported the lowest mortality among light drinkers in a pooled analysis of over 500,000 participants.^[15]^ In our analysis, former drinkers consistently exhibited the highest mortality risk, which may be partially attributed to the “sick quitter” phenomenon, where individuals abstain due to declining health. Although we adjusted for comorbidities, residual confounding likely remains, suggesting the observed protective effect of low-level drinking is not entirely attributable to reverse causation.

In contrast to all-cause mortality, alcohol consumption was not significantly associated with cardiovascular mortality in this cohort. The limited number of cardiovascular deaths may have reduced the statistical power to detect modest associations. Additionally, previous studies have yielded inconsistent findings regarding alcohol and cardiovascular outcomes, particularly in patients with existing cerebrovascular disease.^[16,17]^ It is possible that, among stroke survivors, cardiovascular risk is more strongly determined by established factors such as hypertension, diabetes, and medication adherence rather than by variations in alcohol intake.^[18,19]^ The RCS analysis further supported this interpretation, showing minimal separation between survival curves and wide CIs across all drinking levels.

A novel aspect of this study was the use of formal mediation analysis to assess whether systemic inflammation could explain the link between alcohol intake and mortality. Although prior studies have linked chronic alcohol use to immune alterations and leukocyte dysregulation,^[20,21]^ we found no evidence that the examined biomarkers (NLR, PLR, NPAR, WBC) acted as significant mediators. Even in combined models, the proportion mediated remained below 10%, and all indirect effects were nonsignificant. These findings suggest that alternative mechanisms – such as vascular endothelial modulation, lipid profile alterations, or psychosocial benefits of light drinking – may underlie the survival advantage.

This dissociation between a clear survival benefit and the absence of inflammatory mediation underscores the complexity of alcohol’s biological effects. Inflammatory status is a known predictor of poor prognosis after stroke, but it may not explain the differential mortality across alcohol intake categories. This highlights the complexity of alcohol-related effects in clinical populations and supports the need for more nuanced investigation of underlying biological mechanisms using longitudinal designs and a broader array of biomarkers.

A major strength is the incorporation of both a large, representative sample and clearly defined mortality outcomes, and rigorous adjustment for confounding variables. Nonetheless, several limitations must be considered. Due to the observational nature of the study, causal inference cannot be established. In addition, although NHANES uses a complex, multistage sampling design, unweighted analyses were applied because weighting in small subgroups may produce unstable variance estimates; therefore, the generalizability of the findings to the broader U.S. population may be limited. Alcohol consumption was self-reported and assessed only once, which may have introduced recall bias or misclassification. Inflammatory biomarkers were also measured at a single time point, which limits the ability to capture dynamic immune changes over time. Finally, the mediation analysis focused on 4 selected markers and may not reflect the full spectrum of immunological or metabolic pathways involved.

## 6. Conclusion

Light-to-moderate alcohol consumption was statistically associated with lower all-cause mortality among stroke survivors, whereas no significant association was found for cardiovascular mortality. Systemic inflammatory markers did not show meaningful mediation of these relationships. These results represent observational associations derived from the study population.

## Author contributions

**Conceptualization:** Xiaoxiu Zhu, Qianqian Zhang.

**Data curation:** Xiaoxiu Zhu, Qianqian Zhang.

**Formal analysis:** Xiaoxiu Zhu, Qianqian Zhang.

**Funding acquisition:** Xiaoxiu Zhu, Qianqian Zhang.

**Investigation:** Xiaoxiu Zhu, Qianqian Zhang.

**Methodology:** Xiaoxiu Zhu, Qianqian Zhang.

**Project administration:** Xiaoxiu Zhu, Qianqian Zhang.

**Resources:** Xiaoxiu Zhu, Qianqian Zhang.

**Software:** Xiaoxiu Zhu, Qianqian Zhang.

**Supervision:** Xiaoxiu Zhu, Qianqian Zhang.

**Validation:** Xiaoxiu Zhu, Qianqian Zhang.

**Visualization:** Xiaoxiu Zhu, Qianqian Zhang.

**Writing – original draft:** Xiaoxiu Zhu, Qianqian Zhang.

**Writing – review & editing:** Xiaoxiu Zhu, Qianqian Zhang.
